# The Nexus of Iron, Senescence, and Fibrosis in Endometriosis: A Narrative Review

**DOI:** 10.1007/s43032-025-01999-0

**Published:** 2025-10-27

**Authors:** Richa Patel, Guruprasad Kalthur, Ratul Datta, Swar Shah, Rahul Dutta

**Affiliations:** 1 Division of Reproductive Biology, Department of Reproductive Science, Kasturba Medical College, Manipal Academy of Higher Education, Manipal, 576104 Karnataka India; 2 Sree Mookambika Institute of Medical Sciences, Kulasekharam, Tamil Nadu India; 3 Nova IVF Fertility, Pantaloons Building, Six Mile, Guwahati, 781036 Assam India; 4 Krishna Nursing Home, 4-D, near Deluxe Bus Stand, Tashkand Society, Nizampura, Gujarat-390002 Vadodara, India; 5 Center for Animal Research, Ethics & Training, Manipal Academy of Higher Education, Manipal, 576104 Karnataka India

**Keywords:** Endometriosis, Cellular senescence, Iron overload, Fibrosis, Inflammation, Therapeutic strategies

## Abstract

Endometriosis is a prevalent chronic inflammatory condition impacting 5–10% of reproductive-age women, commonly resulting in debilitating pelvic pain and infertility. Despite extensive research efforts, the precise underlying pathophysiology remains largely unclear. Emerging evidence increasingly suggests that cellular senescence, iron overload, and fibrosis collectively form a critical pathological axis that significantly contributes to the persistence and severity of the disease. However, the intricate mechanistic interplay between the immune system’s failure to effectively clear senescent cells, the damaging effects of iron-induced oxidative stress, and the subsequent fibrotic remodelling is still poorly understood. This narrative review highlights the interconnected roles of impaired immune clearance of senescent cells, iron accumulation, and fibrosis development in driving endometriosis pathogenesis. The review aims to clarify how iron overload and cellular senescence contribute to the progression of endometriosis. It also evaluates novel therapeutic strategies that target iron dysregulation and senescence pathways. By exploring this detrimental triad, we seek to identify potential new avenues for transforming the management of endometriosis, offering hope for more effective treatments to alleviate the significant burden on affected women.

## Introduction

### The Enigma of Endometriosis

Endometriosis is characterized by endometrium-like tissue (lesion) outside the uterus on the peritoneal lining of the pelvic cavity or the ovaries [1]. The most common locations of ectopic implants are within the pelvis, on the peritoneum, ovaries, fallopian tubes, rectovaginal septum, and uterosacral ligaments [2]. Lesions can also be found in extrapelvic locations such as the bowel, bladder, diaphragm, pleura, pericardium, or even the central nervous system [3]. This etiologically complex, multifactorial inflammatory condition affects ~ 6–10% (190 million) of women of reproductive age worldwide [4]. Endometriosis imposes a significant burden through its primary symptoms: chronic pelvic pain, severe dysmenorrhea, dyspareunia, dyschezia, and infertility [5].

The precise origins and mechanisms driving endometriosis remain incompletely understood, despite decades of research [6]. Several theories have been proposed, including coelomic metaplasia, Müllerian remnants, lymphatic/vascular metastasis, and stem cell involvement [7]. However, the most widely accepted hypothesis is the retrograde menstruation theory. As per this theory, during menstruation, endometrial cells and tissue fragments flow backward through the fallopian tubes and implant onto pelvic surfaces [8]. While retrograde menstruation is observed in the majority of women, only a fraction develop endometriosis [9]. This indicates that other factors, likely involving immune dysfunction, genetic predisposition [10], hormonal imbalances, and environmental influences, are crucial for these ectopic cells’ survival, implantation, and proliferation [11]. Moreover, endometriosis is fundamentally recognized as an estrogen-dependent, chronic inflammatory disease [3].

### Aging and the Female Reproductive System

Aging is a natural process, and efforts to win the race against aging have been a constant human endeavor. The understanding of cellular aging has been rudimentary but is evolving rapidly with the availability of new research-driven data. Biological aging affects all organ systems and is characterized by a progressive decline in function and increased susceptibility to disease [12]. Although all the organ systems age progressively with time, the female reproductive system stands out. It comes with a physiological functional validity characterized by an ever-diminishing ovarian reserve that starts even before birth [13]. The physiological changes associated with female aging affect the overall well-being and quality of life; however, most of the research in this field is skewed towards ovarian aging only.

Traditionally, research on female reproductive aging focuses mainly on the depletion of the finite oocyte pool and the decline in oocyte quality due to factors like increased meiotic errors and aneuploidy [14]. However, evidence suggests that the uterus and the endometrium also undergo significant age-related changes that contribute independently to reproductive decline [15]. From this point of view, the role of aging in uterine and endometrial pathologies has been poorly studied. Recent studies have shown that endometrial cell senescence has been associated with implantation failure [16], impaired decidualization [17], and recurrent pregnancy loss [18]. Studies involving oocyte donation and the transfer of euploid embryos have revealed lower implantation and pregnancy rates. It also highlighted the higher rates of pregnancy loss in older women compared to younger recipients, even when oocyte quality is held constant [19]. This strongly implicates an age-related decline in uterine receptivity and function. Furthermore, the endometrium exhibits accelerated aging at a molecular level compared to other tissues, as measured by epigenetic clocks [19].

### Emerging Concepts in Endometriosis Pathogenesis

Emerging evidence suggests that aging and chronic disease-related processes may play a key role in endometriosis beyond its classical features of ectopic tissue, inflammation, and hormonal dependence. These include cellular senescence, iron overload, and fibrosis.

Cellular senescence, a state of stable cell cycle arrest coupled with a pro-inflammatory secretome, is increasingly recognized for its role in aging and various pathologies [20]. Its potential involvement in endometrial function and dysfunction is gaining attention [21]. The chronic, low-grade inflammation characteristic of endometriosis bears resemblance to “inflammaging,” the systemic increase in inflammation observed with aging [12].

An overlooked factor in the progression of endometriosis is iron overload. Breakdown of erythrocytes from retrograde menstruation or ectopic lesions leads to iron accumulation in the peritoneal cavity [22]. Free iron and ferritin levels have been reported to be significantly higher in the peritoneal fluid of patients with endometriosis compared to healthy controls [23]. This excess iron can catalyze the formation of reactive oxygen species (ROS), leading to oxidative stress and cellular damage [24].

Fibrosis and myofibroblast accumulation are persistent features of endometriotic lesions, making fibrosis a molecular hallmark of endometriosis [25]. Fibrosis contributes to key pathological features of endometriosis, like pain, adhesions, and organ dysfunction [26]. Fibrotic tissue alters local iron homeostasis, leading to iron sequestration and oxidative stress. The chronic accumulation of iron, largely derived from retrograde menstruation and erythrocyte breakdown, further amplifies oxidative damage and inflammation. Iron overload, through ROS production, promotes cellular senescence in surrounding cells, thereby reinforcing a self-sustaining cycle of fibrosis, inflammation, and senescence [27].

### Rationale and Scope of the Review

While inflammation, immune dysfunction, and hormones are well-known in endometriosis, the link between iron overload, senescence, and fibrosis remains largely unexplored. Iron overload can drive oxidative stress, which in turn can induce senescence and fibrosis [27]. Senescent cells create a pro-inflammatory and pro-fibrotic milieu by releasing Senescence-Associated Secretory Phenotype (SASP) [28]. Chronic inflammation itself is known to be a driver of fibrosis [29]. The accumulation of iron-laden macrophages and fibrotic lesions in endometriotic tissue suggests iron overload. This fuels a vicious cycle of oxidative stress, senescence, and fibrosis, exacerbating disease progression. Although inflammation, iron-induced oxidative stress, and senescence are plausibly linked, their molecular interplay in endometriosis is poorly understood. No study has systematically examined whether iron overload drives senescence and fibrosis, or if senescence worsens iron retention and chronic inflammation.

**Fig. 1:**
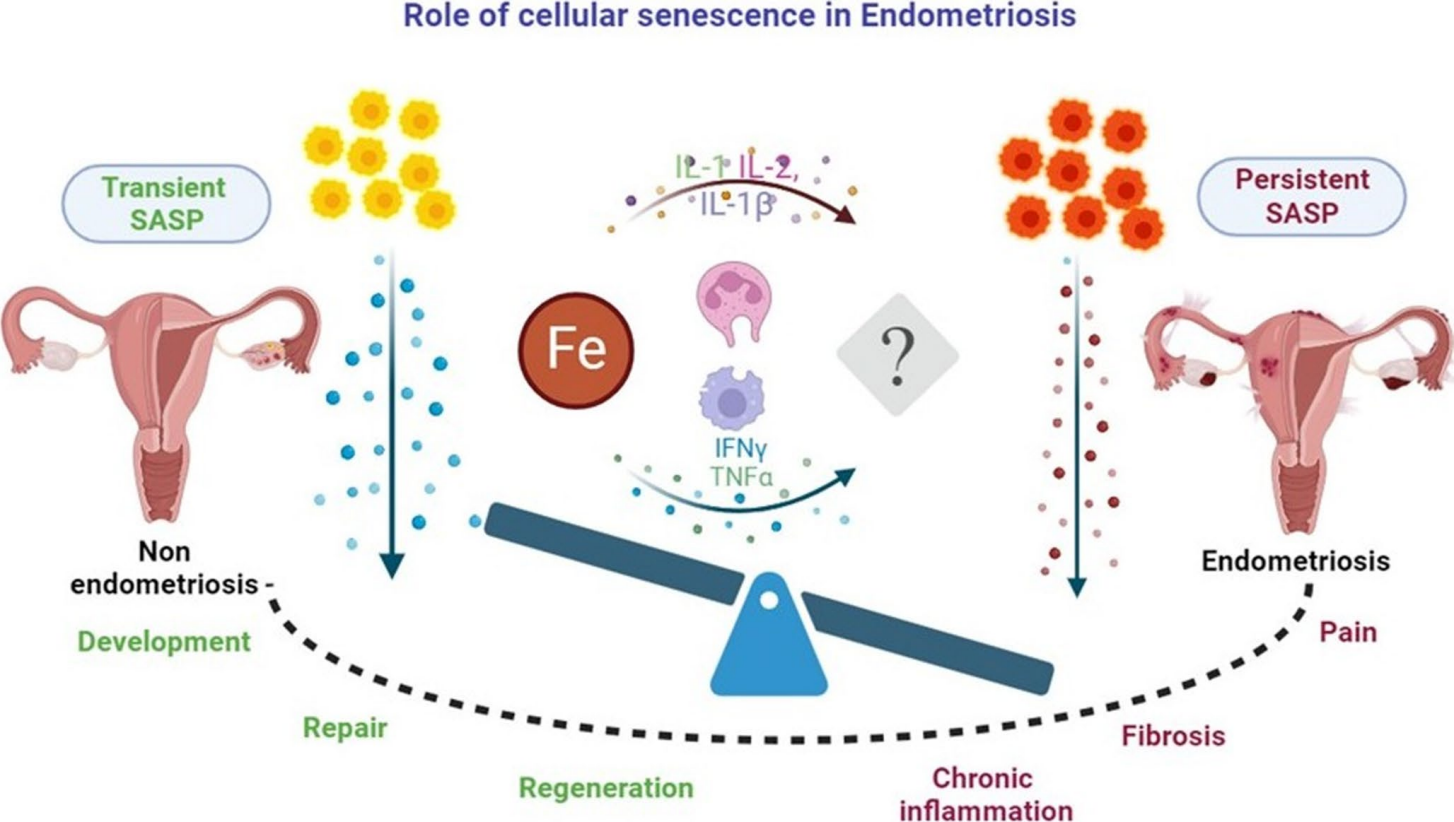
This flowchart outlines the proposed self-perpetuating cycle driving endometriosis. Initial hemorrhage leads to iron overload, causing oxidative stress and inducing cellular senescence. Senescent cells release SASP factors, fuelling chronic inflammation and promoting fibrosis. Immune evasion mechanisms allow these pathological drivers to persist. The cycle is reinforced by feedback mechanisms, including further iron accumulation in senescent cells, impaired clearance due to fibrosis, and recurrent hemorrhage, highlighting the interconnected roles of iron, senescence, and fibrosis in disease progression (Created with BioRender)

This narrative review amalgamates current insights into uterine aging, cellular senescence, iron metabolism, and fibrosis in the context of endometriosis. It highlights emerging evidence of the interconnectedness among iron overload, senescence, and fibrosis, examining their potential crosstalk and collective role in disease progression (Fig. 1). By exploring this triad, the review aims to uncover the vicious cycle driving chronic inflammation and identify novel avenues for therapeutic intervention.

## Method

**Fig. 2:**
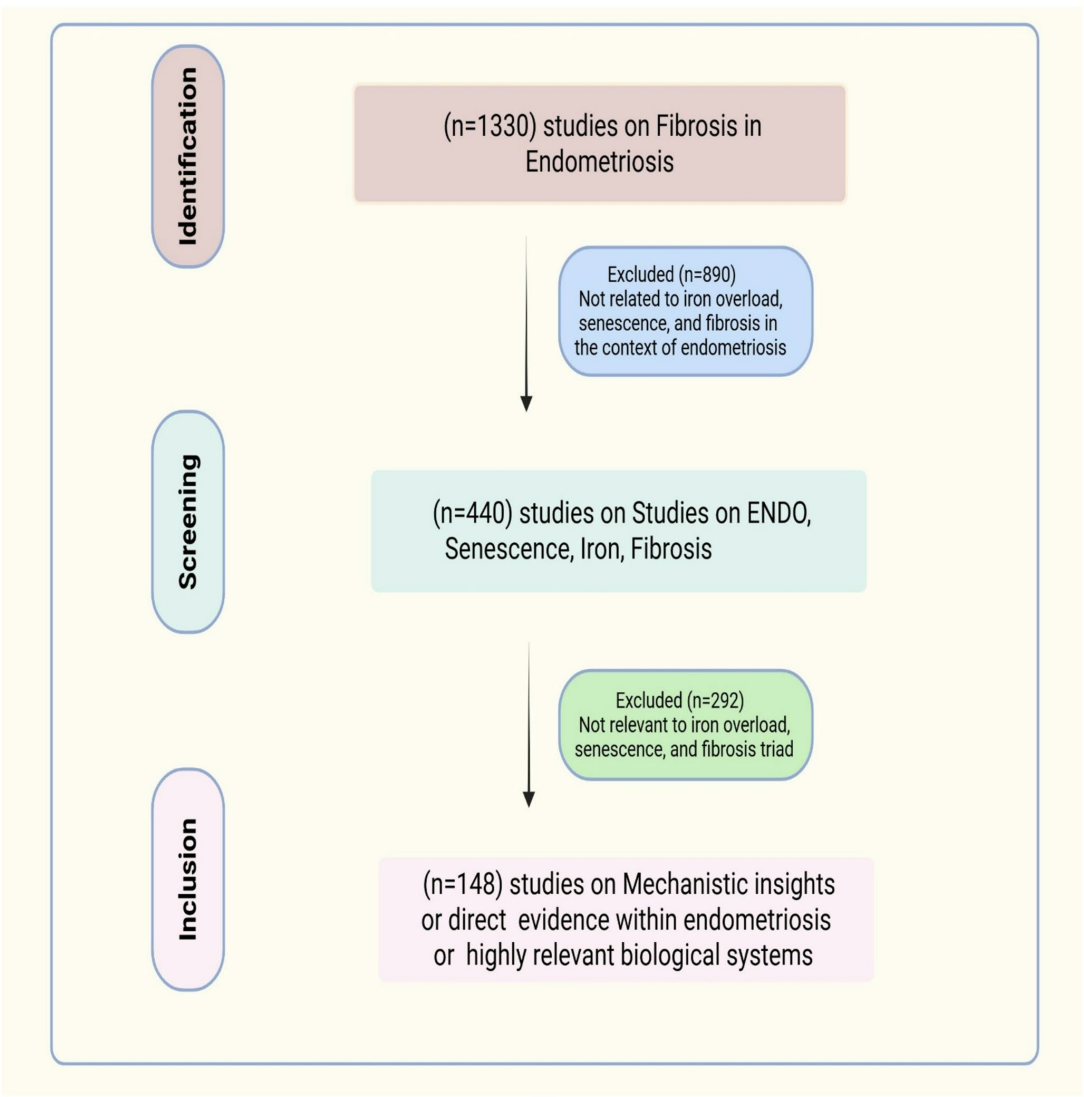
The brief depiction of the process of literature search and screening for the review

To search for articles related to the interplay of Iron, senescence, and fibrosis in endometriosis, the electronic databases PubMed, Google Scholar, and Scopus were searched. The search was performed via the keywords “endometriosis,” “cellular senescence,” “iron overload,” “fibrosis,” “inflammation,” “therapeutic strategies,” “uterine aging,” “SASP,” and combinations thereof. The inclusion criteria were (1) articles that provided insights into the interconnectedness of iron overload, senescence, and fibrosis in the context of endometriosis pathogenesis, (2) either qualitative or quantitative studies, and (3) studies published till 2025. The exclusion criteria were (1) non-English language articles, (2) studies not directly relevant to the proposed triad. The search strategy is presented in detail in the scheme in Fig. 2. The articles were selected based on their relevance, scientific rigor, and contribution to the understanding of the proposed “vicious cycle.” We prioritized studies that provided mechanistic insights or direct evidence within endometriosis or highly relevant biological systems.

## Uterine and Endometrial Aging: the Neglected Component

The contribution of age-related changes within the uterus and endometrium is increasingly recognized as a significant factor affecting fertility and pregnancy outcomes [15]. Despite its regenerative nature, the endometrium remains susceptible to aging [30].

### Age-Related Changes in the Uterus and Endometrium

Advancing maternal age is associated with distinct structural and functional alterations in the uterus and endometrium. Morphologically, the uterus tends to shrink with age [31]. Vascular changes include a reduced blood supply, with narrowing of uterine veins and progressive loss of uterine spiral arteries [19]. Histologically, aging disrupts the normal endometrial architecture and alters its cellular composition [30]. Additionally, hormone production and responsiveness can be disrupted, potentially shortening menstrual cycles. It also impairs the progression of the endometrium to the receptive secretory stage required for implantation [19].

Endometrial thickness decreases in older women, with a meta-analysis showing a reduction of about 0.52 mm in women over 35–40 years compared to younger women [19]. These age-related uterine and endometrial changes reduce the capacity for successful fertilization, embryo implantation, and pregnancy maintenance [13]. Advanced maternal age is linked to a ~ 27% reduced chance of implantation, a ~ 20% lower chance of clinical pregnancy, and a ~ 44% higher risk of pregnancy loss [19]. This applies even when factors such as oocyte quality are accounted for by using donor oocytes or transferring genetically normal embryos. This underscores the critical importance of the uterine environment itself in determining reproductive success, independent of the embryo’s quality. Molecular studies using epigenetic clocks have shown that reproductive tissues, including the endometrium, age at an accelerated rate compared to non-reproductive tissues [32].

## Molecular Mechanisms of Uterine/Endometrial Aging

The functional decline observed in the aging uterus and endometrium is underpinned by alterations at the molecular level, implicating several key pathways [15]. Oxidative stress, caused by an imbalance between ROS production and antioxidant defences, is a key contributor to aging [33]. Dysregulation of inflammatory and immune responses is also evident, often shifting towards a more pro-inflammatory state with altered cytokine profiles [34]. Compromised mitochondrial function affects energy production and processes like myometrial contractility [35]. Alterations in DNA damage response pathways [36], mTOR signaling [37], the endocannabinoid system [38], and the SIRT1 longevity [39] pathway have also been observed in aging uterine tissues. Furthermore, age-related decline of estrogen receptors in the endometrial stroma can impair tissue responsiveness [40].

Specific molecular changes within the aging endometrium include altered gene expression profiles. In women over 35, genes related to cilia motility and ciliogenesis are upregulated, possibly as a compensatory response or due to downregulation of epithelial cell proliferation [41]. Dysregulation of the VEGF signaling pathway, vital for angiogenesis, occurs alongside inhibited epithelial proliferation, possibly driven by genes involved in cell cycle arrest and telomere protection [41]. Decreased proliferation of menstrual blood stem cells has also been reported [42]. Signaling pathways like the Sonic Hedgehog (SHH) pathway show declining activity in aging endometrial stem cells [15]. Epigenetic modifications, such as increased methylation and expression of the pseudogene PTENP1, occur with age, possibly as a protective mechanism against malignancy [43]. Additionally, changes in the expression of pro-inflammatory cytokines (e.g., Il17RB) [44] and chemokines (e.g., CXCL12, CXCL14) [44], as well as specific microRNAs (e.g., miR-223-3p, 155-5p, 129-5p in mice) [45], have been identified as potential biomarkers of endometrial aging.

These molecular shifts suggest that uterine aging is not simply a passive deterioration but involves active reprogramming that directly impacts tissue function. Accelerated epigenetic aging in the endometrium may indicate a heightened vulnerability to age-related defects, potentially predisposing the tissue to dysfunction or conditions like endometriosis earlier than expected [32].

### Cellular Senescence in Endometrial Aging

Cellular senescence, the process of irreversible growth arrest often triggered by stress or damage, is increasingly recognized as a contributor to uterine and endometrial aging [46]. Senescence markers, including the cell cycle inhibitors p21 and p53, and SA-β-Gal activity, have been observed in aging endometrial cells, particularly within the stromal compartment [30]. Studies comparing endometrial stromal cells from younger and older women (>36 years) show age-related reductions in stromal cell proliferation and lower expression of key regulators like BMP2 and STAT3 [47]. Furthermore, upon in vitro decidualization, stromal cells from older women exhibit significantly lower expression of crucial decidual markers such as prolactin (PRL) and insulin-like growth factor-binding protein-1 (IGFBP-1), indicating impaired functional capacity with age [47].

This age-associated increase in endometrial senescence is not merely a benign marker of time but seems to facilitate adverse reproductive outcomes. Endometrial stromal cell senescence has been linked to conditions like recurrent implantation failure (RIF) and recurrent pregnancy loss (RPL) [30]. This establishes a plausible link between aging-related cellular processes in the endometrium and reproductive pathologies.

## Cellular Senescence: A Double-Edged Sword

Cellular senescence is a fundamental biological process characterized by a stable and essentially irreversible cell cycle arrest in cells that were previously capable of proliferation. Originally, it was described as an irreversible loss of proliferation in cells with a possible intrinsic antitumor barrier function. Physiologically, the timely and localized presence of senescent cells is vital for wound healing, tissue repair, and regeneration [48]. It serves as a crucial stress response mechanism, limiting the propagation of damaged or potentially cancerous cells [20].

### Hallmarks of Senescence

Senescence can be triggered by a variety of intrinsic and extrinsic factors. These include replicative exhaustion due to telomere shortening, DNA damage, oncogene activation, mitochondrial dysfunction, metabolic stress, proteotoxic stress, and exposure to certain inflammatory mediators or mitogens [49].

Regardless of the trigger, senescent cells share a set of core features. The defining characteristic is the durable growth arrest, typically enforced by the activation of tumor suppressor pathways involving key cell cycle inhibitors like p16^Ink4a^(encoded by *CDKN2A*) and p21^Cip1^ (encoded by *CDKN1A*), often acting via p53 and the retinoblastoma protein (pRB) [50, 51]. Senescent cells undergo distinct morphological changes, often becoming larger, flatter, and exhibiting alterations in organelles like lysosomes and mitochondria [52]. They also display significant chromatin remodeling, forming senescence-associated heterochromatin foci (SAHF), and profound changes in gene expression [49]. Despite their inability to divide, senescent cells remain metabolically active. They develop resistance to apoptosis (programmed cell death), partly through the upregulation of senescent cell anti-apoptotic pathways (SCAPs) [53]. Common biomarkers used to identify senescent cells include the expression of p16^INK4a^ and p21^Cip1^, increased activity of SA-β-gal at pH 6.0, loss of the nuclear envelope protein Lamin B1, and the presence of persistent DNA damage foci [54].

### The Senescence-Associated Secretory Phenotype (SASP)

One of the most impactful features of senescent cells is their acquisition of a complex secretome known as the SASP [55]. The SASP consists of a plethora of secreted factors, including pro-inflammatory cytokines, chemokines, growth factors, and extracellular matrix-degrading proteases, which profoundly influence the tissue microenvironment [56].

Key components of the SASP include:

- **Pro-inflammatory Cytokines**: Interleukin-6 (IL-6) is a hallmark SASP factor, along with IL-1α, IL-1β, IL-8 (CXCL8), IL-7, IL-13, IL-15 and Tumor Necrosis Factor-alpha (TNF-α) [55].
- **Chemokines**: Various chemokines attracting immune cells are secreted, such as IL-8 (CXCL8), GROα/β (CXCL1/2), MCP-1 (CCL2), MCP-2 (CCL8), RANTES (CCL5), MIP-1α (CCL3), and SDF-1 (CXCL12) [57].
- **Growth Factors**: Factors promoting cell growth and angiogenesis, like VEGF, can be part of the SASP [57].
- **Proteases**: Enzymes that remodel the ECM are characteristic, including Matrix Metalloproteinases (MMPs) such as MMP-1 (collagenase), MMP-3 (stromelysin-1), MMP-10 (stromelysin-2), and MMP-12. Serine proteases like urokinase-type plasminogen activator (uPA) and tissue-type plasminogen activator (tPA), along with their inhibitors (PAI-1, PAI-2) and other proteases like Cathepsin B, are also secreted [57].

The composition of the SASP is not fixed but varies depending on the cell type, the senescence-inducing stimulus, and the duration of senescence [58]. This context-dependency implies that the specific effects of senescence might differ across various tissues and diseases. For example, SASP profiles from iron-induced senescent endometrial cells in endometriosis may differ from those in fibroblasts undergoing telomere-related senescence. Understanding the specific SASP in endometriosis is therefore crucial. The SASP is regulated by complex signaling networks, often involving transcription factors like NF-κB and C/EBPβ, and can be reinforced by autocrine feedback loops involving factors like IL-1α [20].

### Physiological vs. Pathological Roles

Cellular senescence plays a paradoxical role in biology, exhibiting beneficial and detrimental functions. Its primary beneficial role is tumor suppression, acting as a barrier to prevent the proliferation of damaged or oncogene-activated cells [59]. Additionally, transient senescence plays a crucial role during embryonic development, facilitating tissue remodeling and morphogenesis [60]. It is also involved in physiological tissue repair and wound healing processes. These beneficial effects depend on senescence being acute and transient, with senescent cells cleared by the immune system, such as macrophages or NK cells, after fulfilling their role [61].

However, when senescent cells evade clearance and accumulate chronically, as often occurs with aging or in pathological conditions, their persistent SASP secretion becomes detrimental [55]. Chronic SASP creates a pro-inflammatory environment, disrupts tissue homeostasis, promotes dysfunction and fibrosis, and may even drive cancer progression [62]. This chronic accumulation of senescent cells is considered a key driver of organismal aging and a major contributor to the pathogenesis of numerous age-related diseases [12].

The endometrium undergoes cyclical remodeling, with programmed and temporary senescence playing a key role in processes like decidualization. Therefore, distinguishing between transient, beneficial senescence and chronic, pathological senescence is especially important in this tissue [16]. A failure in the mechanisms responsible for clearing these transiently senescent cells (e.g., dysfunction of uterine NK cells) could lead to their persistence and accumulation. This may transform a physiological process into a pathological driver of chronic inflammation and tissue dysfunction, potentially contributing to diseases like endometriosis (Table 1).

**Table 1:** Key characteristics and markers of cellular senescence

**Feature**	**Description**	**Key Molecular Markers/Indicators**	**Reference**
Cell cycle arrest	Stable, essentially irreversible exit from the cell cycle; resistance to mitogenic stimuli.	↑ p16^INK4a^,(*CDKN2A*), ↑ p21^Cip1^(*CDKN1A*), ↑ p53; Increased G0/G1 phase population.	[30]
Morphological changes	Increased cell size, flattened morphology, altered organelle structure (lysosomes, mitochondria).	Often observed microscopically.	[55]
SA-β-Gal activity	Increased lysosomal β-galactosidase activity detectable at suboptimal pH (pH 6.0).	Positive staining with X-Gal substrate at pH 6.0.	[20]
Chromatin remodeling	Formation of Senescence-Associated Heterochromatin Foci (SAHF); altered gene expression.	Visible foci (in some contexts); changes in histone modifications.	[20]
Apoptosis resistance	Upregulation of anti-apoptotic pathways (SCAPs) allowing survival despite pro-apoptotic SASP factors.	↑ Expression of SCAP pathway components (e.g., specific BCL-2 family members).	[54]
Lamin B1 loss	Downregulation or loss of nuclear lamina protein Lamin B1.	Decreased Lamin B1 staining via immunofluorescence/immunohistochemistry.	[55]
SASP secretion	Secretion of a complex mix of pro-inflammatory cytokines, chemokines, growth factors, and proteases.	↑ Secretion/expression of IL-6, IL-8, TNF-α, CCL2, MMP-1, MMP-3, PAI-1, etc.	[56, 58]
Metabolic alterations	Changes in cellular metabolism, often increased glycolysis and mitochondrial dysfunction.	Altered metabolite levels, changes in metabolic enzyme expression.	[20]

## Endometriosis Pathophysiology: Inflammation, Immunity, and Fibrosis

The pathophysiology of endometriosis is complex, involving an interplay of hormonal influences, genetic predisposition, and environmental factors. Central to the establishment and persistence of the disease are chronic inflammation, immune system dysfunction, and progressive tissue remodeling, including fibrosis [3].

### The Inflammatory Milieu

Endometriosis is fundamentally a chronic inflammatory condition [63]. The peritoneal cavity of women with endometriosis harbors a distinct inflammatory milieu compared to healthy controls. Peritoneal fluid analysis consistently reveals an increased number and activation state of macrophages [64]. This is accompanied by elevation in the levels of various pro-inflammatory cytokines and chemokines, including TNF-α, IL-1β, IL-6, IL-8, RANTES (CCL5), and Monocyte Chemoattractant Protein-1 (MCP-1) [65–68]. Prostaglandins, particularly PGE_2_ and PGF_2α_, are abundant, often produced by activated peritoneal macrophages expressing high levels of cyclo-oxygenase-2 (COX-2) [69].

These inflammatory mediators are not merely bystanders but actively contribute to disease pathogenesis. Cytokines like IL-8 and TNF-α promote the proliferation and adhesion of ectopic endometrial cells and stimulate angiogenesis necessary for lesion survival and growth [70]. Chemokines such as RANTES and MCP-1 recruit more immune cells, particularly macrophages, to the sites of inflammation, potentially perpetuating the cycle [68]. Mediators like PGE_2_ can stimulate local estrogen production within lesions by activating enzymes like aromatase, further driving the estrogen-dependent nature of the disease [71]. Key inflammatory signaling pathways, such as the NF-κB pathway, are often activated in endometriotic tissues, driving the expression of many of these inflammatory molecules [72]. Evidence suggests that the eutopic endometrium of women with endometriosis also exhibits inflammatory changes, such as increased macrophage numbers and elevated basal IL-6 production [73, 74]. This potentially causes implantation problems.

The specific profile of inflammatory mediators dominant in endometriosis (high IL-1β, IL-6, IL-8, TNF-α, CCL2/MCP-1, MMPs) shows a striking resemblance to the canonical components of the SASP secreted by senescent cells [57]. This overlap strongly supports the notion that cellular senescence may be a significant contributor to, or even a source of, the chronic inflammation observed in endometriosis.

### Immune System Dysfunction

A critical factor enabling the establishment and persistence of endometriosis is a failure of the immune system to effectively clear the refluxed endometrial tissue from the peritoneal cavity [75]. Normally, immune cells like NK cells and macrophages would recognize and eliminate this misplaced tissue. However, various reports have shown that the peripheral blood and local environment resident NK cell population in endometriosis is dysregulated with reduced cytotoxic ability [76, 77]. This reduced killing capacity may allow ectopic endometrial cells to evade immune surveillance and survive in the peritoneum [78]. Potential mechanisms underlying this NK cell defect include altered expression of activating and inhibitory receptors on the NK cell surface (favoring inhibition) and potentially suppressive factors within the peritoneal fluid [79]. Furthermore, eutopic endometrial cells from women with endometriosis may be more resistant to NK cell lysis, possibly due to shedding of adhesion molecules like ICAM-1 [3].

Macrophage function also appears to be impaired. While their numbers are increased in the peritoneal fluid, their phagocytic capacity may be reduced [80]. This could further contribute to the inefficient clearance of endometrial fragments. Alterations in other immune cell populations, including T lymphocytes (with potential shifts in subsets like T-helper and T-regulatory cells), B lymphocytes, dendritic cells, and neutrophils, have also been reported, contributing to a complex dysregulation of the local immune microenvironment [81]. This overall immune dysfunction not only facilitates the establishment of lesions but also contributes to a chronic inflammatory state.

The observed immune defects, particularly the dysfunction of NK cells, may have broader implications beyond simply allowing ectopic tissue survival. Since NK cells help clear senescent cells, their impaired function in endometriosis may hinder this process [82]. This impairment in NK cell function has a two-fold pathological effect within the proposed disease triad. The primary role of NK cells is to recognize and eliminate misplaced endometrial cells that reflux into the peritoneal cavity. When this function is compromised, ectopic cells can evade immune surveillance, survive, and implant. Beyond this, NK cells are also crucial for clearing physiologically senescent cells promptly. The established dysfunction of NK cells in endometriosis means they may also fail in this surveillance role, allowing senescent cells to persist and accumulate within the endometriotic lesions and the surrounding microenvironment. This dual failure to clear both ectopic tissue and senescent cells creates a permissive environment for the chronic inflammation that characterizes endometriosis. The accumulation of senescent cells leads to a buildup of their pro-inflammatory SASP, which, combined with the presence of viable ectopic tissue, drives the vicious cycle of inflammation, fibrosis, and disease progression.

### Fibrosis as a Pathological Hallmark

Fibrosis, leading to scarring, adhesions, and tissue hardening, is increasingly appreciated as a fundamental pathological feature of endometriosis [26]. This fibrotic process contributes significantly to the clinical burden of the disease, particularly chronic pain and infertility [26]. Fibrosis is observed in various forms of endometriosis, including superficial peritoneal lesions, ovarian endometriomas, and deep infiltrating endometriosis (DIE), where it is particularly prominent and contributes to invasion into surrounding organs [83]. Some researchers have even proposed that endometriosis should be redefined primarily as a fibrotic condition, highlighting the centrality of this process [84].

The key cellular mediators of fibrosis are myofibroblasts, specialized cells characterized by expression of α-SMA and a high capacity for producing and depositing ECM proteins, primarily collagen types I and III [85]. These cells can arise from various sources, including the transdifferentiation of resident fibroblasts (fibroblast-to-myofibroblast transition, FMT), epithelial cells (epithelial-to-mesenchymal transition, EMT), or endothelial cells (endothelial-to-mesenchymal transition, EndMT) [86]. TGF-β is recognized as a master regulator driving myofibroblast differentiation and ECM production in endometriosis, similar to its role in other fibrotic diseases [87]. The persistent inflammation and repetitive tissue injury associated with cyclical bleeding from endometriotic lesions possibly provide the chronic stimuli that drive sustained myofibroblast activation and progressive fibrosis [88].

## Iron Overload: Fuelling the Fire in Endometriosis

An often-overlooked factor in endometriosis pathogenesis is the dysregulation of iron metabolism, leading to iron overload within the pelvic microenvironment [22]. Studies point towards a significant role for excess iron in driving key pathological processes of the disease.

### Evidence of Iron Dysregulation

Numerous studies have consistently demonstrated elevated levels of iron markers in women with endometriosis compared to controls without the disease. Specifically, increased free iron concentration, the iron storage protein ferritin, and higher transferrin (Tf) saturation have been detected in peritoneal fluid, the fluid within ovarian endometriomas, ectopic lesion tissue, and even within peritoneal macrophages [89]. Ferritin, in particular, is often significantly upregulated, reflecting increased iron storage, potentially in response to the overload [23].

The primary source of this excess iron is from the recurrent hemorrhage associated with endometriosis, both from retrograde menstruation and from bleeding within the ectopic lesions [90]. As RBCs break down, they release large amounts of hemoglobin, which is subsequently degraded, liberating heme and free iron into the microenvironment [24]. Peritoneal macrophages play a key role in phagocytosing these senescent or damaged erythrocytes. However, in the context of chronic bleeding and potential macrophage dysfunction, the release of iron can overwhelm normal storage and handling mechanisms, leading to iron overload [24]. Some studies have reported a correlation between the extent of iron overload (e.g., higher peritoneal fluid iron or ferritin levels) and the severity of endometriosis (e.g., Stage III/IV vs. Stage I/II) [91, 92], although this finding is not universal across all studies [23].

**Fig. 3:**
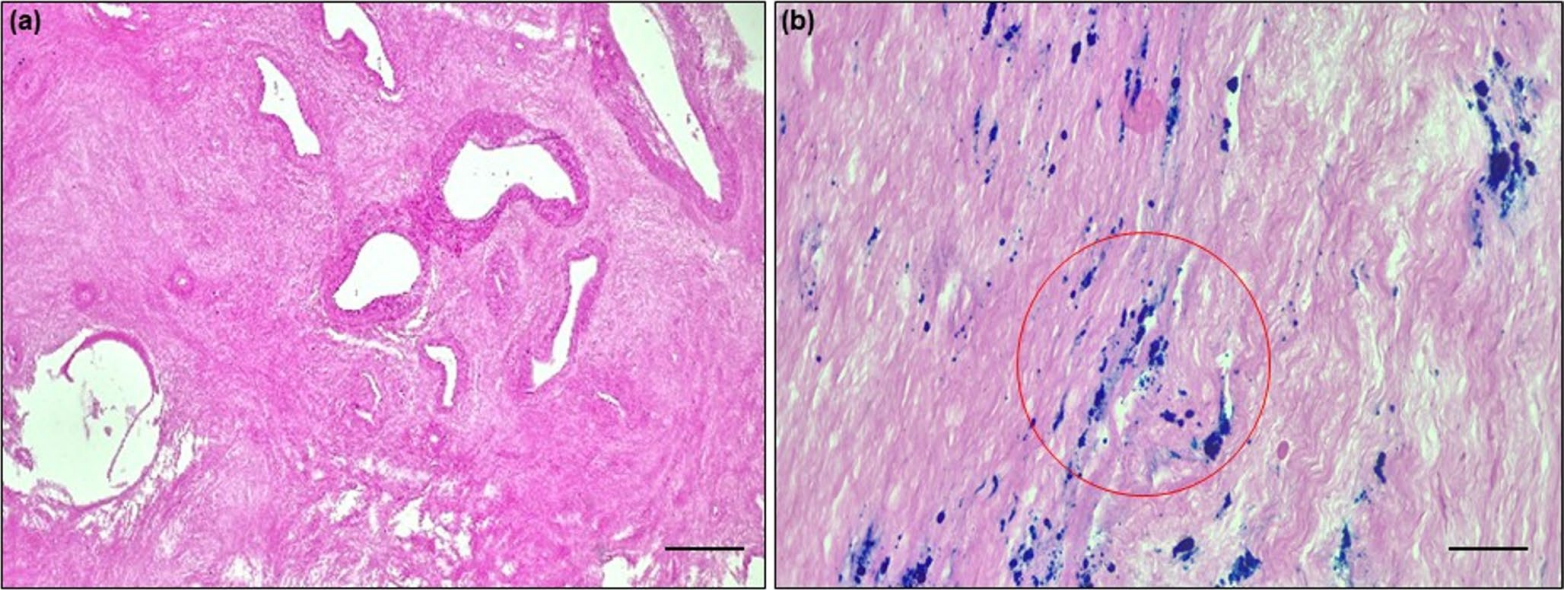
Representative images of Perl’s Prussian Blue staining. (**a**) The peritoneum of a patient without endometriosis (control) displayed no presence of iron (**b**) endometriotic lesion of a stage 4 endometriosis patient indicates high iron presence (blue spots) [unpublished data]

The consistent presence of iron overload across multiple pelvic compartments strongly implicates recurrent bleeding as a key contributor. However, retrograde menstruation is common, while significant iron overload appears more specific to endometriosis. This suggests defects in local iron handling and clearance mechanisms, potentially linked to the immune dysfunction, are likely crucial for the persistence of this overload state [24]. Figure 3 shows the evidence of iron accumulation in human stage 4 endometriotic lesion.

### Mechanisms of Iron-Induced Damage

Iron becomes highly toxic when in excess, primarily due to its ability to catalyze the generation of reactive oxygen species (ROS) [93]. The key reaction is the Fenton reaction, where ferrous iron (Fe^2+^) reacts with hydrogen peroxide (H_2_O_2_) to produce highly reactive and damaging hydroxyl radicals (^−^OH) [94].

Under normal conditions, iron in circulation is safely bound to the transport protein Tf, and intracellular iron is largely sequestered within the storage protein ferritin [95]. However, in iron overload states, the binding capacity of Tf becomes saturated. This leads to the appearance of potentially toxic non-transferrin-bound iron (NTBI) in the plasma and extracellular fluids [96]. Similarly, intracellular excess iron can overwhelm ferritin storage capacity, increasing the size of the labile iron pool (LIP), a pool of loosely bound, redox-active iron within the cell [96]. Both NTBI and an expanded LIP readily participate in the Fenton reaction, driving ROS production [96].

The resulting surge in ROS leads to oxidative stress, a state where the production of oxidants overwhelms the cell’s antioxidant defense systems [97]. This oxidative stress causes widespread damage to cellular components, including lipid peroxidation, protein oxidation, and DNA damage [22].

### Consequences of Iron Overload in Endometriosis

The consequences of iron overload and the resulting oxidative stress are manifold and appear central to endometriosis pathophysiology:

- **Oxidative Stress**: Iron overload is considered a primary driver of the heightened oxidative stress observed in the peritoneal fluid, follicular fluid, and tissues of women with endometriosis [98]. This is supported by findings of increased lipid peroxidation products e.g., malondialdehyde (MDA), 4-hydroxynonenal (4-HNE) and decreased levels or activity of antioxidant enzymes like superoxide dismutase (SOD) and glutathione peroxidase (GPx) in affected women [92].
- **Inflammation**: Iron-induced oxidative stress can directly trigger and amplify inflammation. ROS can activate key pro-inflammatory signaling pathways, most notably NF-κB [72]. Activation of NF-κB in endometrial stromal cells leads to increased expression and secretion of inflammatory mediators like ICAM-1, contributing to the pro-inflammatory microenvironment [92]. This provides a direct molecular pathway linking iron accumulation to the chronic inflammation seen in endometriosis and potentially to the generation of SASP factors, as NF-κB is a major regulator of SASP gene expression [54].
- **Altered Cell Behavior**: Iron overload appears to directly influence the behavior of endometriotic cells, although findings are complex. Some studies, particularly in animal models, suggest that iron overload enhances the proliferation of ectopic endometrial cells, contributing to lesion growth [24]. Conversely, at least one in vitro study reported that high iron concentrations inhibited the proliferation of primary endometrial stromal cells [99]. Concerning endometriosis, iron may have the downstream consequences like inflammation, fibrosis, rather than the initial cell loss itself. More consistently, iron overload has been shown to stimulate the migration and invasion of human endometriotic cells, potentially by upregulating MMPs and inducing EMT processes crucial for lesion establishment and spread [100]. Furthermore, the role of iron in ferroptosis is particularly intriguing and seemingly paradoxical in endometriosis. While high iron levels would typically induce ferroptosis, ectopic endometrial cells may possess mechanisms to resist this process, allowing them to survive and thrive in the iron-rich pelvic environment [101]. However, other studies suggest that iron overload does induce ferroptosis in endometriotic stromal cells or specific subpopulations (like mesenchymal stem cells), and that this process, rather than eliminating cells, might paradoxically promote inflammation and fibrosis [94]. This discrepancy likely reflects context-dependent effects or heterogeneity within endometriotic lesions. The resistance might be crucial for initial cell survival, while later ferroptosis induction in some cells could drive disease progression through inflammatory signaling and fibrotic responses.
- **Infertility/Embryotoxicity**: Iron overload in the pelvic cavity and follicular fluid is strongly linked to endometriosis-associated infertility [102]. Excess iron and the resulting oxidative stress can directly damage oocytes, sperm, and developing embryos, impairing fertilization and early development [102]. Iron overload in follicular fluid has been specifically shown to trigger ferroptosis in granulosa cells, which impairs oocyte maturation [103].
- **Fibrosis**: Emerging evidence directly links iron overload to the development of fibrosis in endometriosis [94]. As mentioned, iron-induced ferroptosis in certain stromal cell populations may trigger fibrotic responses [94] (Table 2).

**Table 2:** Evidence for iron overload in endometriosis compartments

Compartment	Iron Marker(s) Elevated	Key Findings	Reference
Peritoneal fluid (PF)	Free iron, ferritin, transferrin saturation, hemoglobin	Consistently higher levels in women with endometriosis vs. controls. Some studies correlate levels with disease severity (Stage III/IV >I/II). Potential source of iron impacting pelvic organs.	[22, 104]
Ovarian endometrioma (fluid/tissue)	Free iron, ferritin, total iron, hemosiderin	High concentrations found within cysts, often higher in older (more fibrotic) cysts. Suggests local hemorrhage and iron accumulation within the lesion. Linked to ferroptosis and fibrosis.	[86]
Ectopic endometriotic lesions (peritoneal, deep)	Iron deposits (hemosiderin), ferritin, total iron	Iron deposition (often visible histologically as hemosiderin) is characteristic. Ferritin upregulated. Iron overload is linked to proliferation in models.	[105]
Peritoneal macrophages	Increased iron storage (ferritin, hemosiderin)	Macrophages in endometriosis patients show significantly higher iron content, correlating with PF iron levels. Reflects phagocytosis of erythrocytes and potential dysfunction in iron handling.	[106]
Follicular fluid	Iron, ferritin	Elevated levels found in women with endometriosis, particularly ovarian endometriomas. Linked to oxidative stress, granulosa cell ferroptosis, and impaired oocyte quality/maturation.	[107]

## Cellular Senescence: A Conductor of Endometriosis Pathogenesis?

Cellular senescence, initially identified as a tumor suppressor mechanism and contributor to aging, is emerging as a potentially significant player in the pathogenesis of endometriosis. Its pro-inflammatory secretome and complex roles in tissue remodeling align well with key features of the disease.

### Senescence in Endometriotic Tissues

Direct evidence indicates the presence of senescent cells within the endometriotic microenvironment. Studies have detected increased expression of senescence markers, including the cell cycle inhibitors p^16INK4a^ and p21^Cip1^, reduced levels of Lamin B1, and positive staining for SA-β-Gal activity in endometriotic lesions or stromal cells derived from these lesions or patients with endometriosis [105]. Furthermore, components of the SASP, such as IL-6 and IL-8, are found at elevated levels in endometriotic cells or their secretions [57]. Genetic approaches, like Mendelian randomization studies, have also suggested potential causal links between genetic variants associated with cellular aging pathways and the risk of developing endometriosis [106].

The presence of senescence markers within lesions suggests that senescence is not merely a consequence of the patient’s overall chronological age but is likely induced locally within the disease environment [54]. Several factors prevalent in endometriosis could act as senescence triggers. Oxidative stress, driven significantly by iron overload, is a potent inducer of senescence, often via DNA damage pathways [108]. The chronic inflammatory milieu itself, rich in cytokines like IL-1β, can also directly induce or accelerate senescence in endometrial stromal cells [57]. Thus, the unique conditions within the endometriotic lesion, high iron, ROS, and persistent inflammation, likely converge to promote cellular senescence.

### SASP and Chronic Inflammation in Endometriosis

The SASP provides a compelling mechanistic link between cellular senescence and the chronic inflammation characteristic of endometriosis. As previously noted, there is a remarkable overlap between the typical pro-inflammatory cytokines and chemokines secreted by senescent cells (IL-1β, IL-6, IL-8, TNF-α, CCL2, CCL5, etc.). Additionally, their levels are elevated in the peritoneal fluid and lesions of women with endometriosis. This suggests that the SASP emanating from senescent cells within or surrounding the lesions significantly contributes to establishing and maintaining the persistent, non-resolving inflammatory state [57].

The SASP can exert its effects through both autocrine and paracrine mechanisms. Paracrine signaling can induce senescence in adjacent healthy cells, a phenomenon known as the “bystander effect”. This amplifies and propagates the senescent phenotype and its associated inflammation throughout the tissue [57]. This self-reinforcing loop could explain the chronic and often progressive nature of inflammation in endometriosis.

### Senescence, Decidualization, and Endometriosis-Related Infertility

The role of senescence in the endometrium is complex, exhibiting a duality critical for understanding its impact on fertility. Acute, transient senescence is now recognized as a necessary physiological process during the menstrual cycle, particularly for successful decidualization required for embryo implantation [16]. Senescent decidual cells emerge during this process and contribute, via their transient SASP, to tissue remodeling and creating a receptive environment [16].

However, dysregulation of this process, leading to excessive or persistent senescence, is associated with adverse reproductive outcomes. Increased stromal cell senescence has been linked to impaired decidualization, RIF, and RPL [109]. For example, a deficiency in proteins like CDC42 can induce premature senescence in endometrial stromal cells, leading to defective decidualization and fibrosis, phenotypes observed in RIF patients [21].

In the context of endometriosis-associated infertility, the pathological senescence induced by local inflammation and oxidative stress could disrupt normal endometrial function [57]. The persistent SASP from senescent cells within the eutopic or ectopic endometrium might directly impair the decidualization process. This may create an environment hostile to embryo implantation and development. This provides a potential mechanism linking the inflammatory and senescent aspects of endometriosis directly to its impact on fertility. The exact criteria for definitively distinguishing these two states in endometriosis are still an active area of research. Based on the current literature, important molecular markers and functional characteristics that could help differentiate the physiological and pathological stages are summarized in Table 3.

**Table 3:** Summary of salient features of physiological and pathological senescence processes associated with endometriosis

**Feature/Marker**	**Physiological Transient Senescence (Normal Endometrium)**	**Pathological Persistent Senescence (Endometriotic Tissue)**	**References**
Location of senescence	Mainly in the decidua during the implantation window.	Primarily within endometriotic lesions (superficial peritoneal, ovarian, deep infiltrating) and potentially in the eutopic endometrium of affected women.	[54, 106, 108, 110–112]
Senescence-inducing stimuli	Programmed physiological signals during the menstrual cycle.	Chronic exposure to iron overload, reactive oxygen species (ROS), oxidative stress, chronic inflammation, and immune dysfunction.	[106, 110–114]
Cellular senescence markers	Transient expression of p21. Minimal or undetectable levels of SA-β-gal, p16INK4a, and Lamin B1.	Increased expression of cell cycle inhibitors like p16INK4a, p21Cip1, and SA-β-gal activity. Reduced levels of Lamin B1. Elevated levels of mitochondrial ROS.	[108, 110]
SASP (senescence-associated secretory phenotype)	Releases pro-regenerative cytokines (e.g., IL-1, IL-2, IL-1β). Transient contribution to tissue remodeling and a receptive environment.	Chronically secretes pro-inflammatory, pro-fibrotic, and pro-proliferative factors, including IFNγ, TNF-α, IL-1β (chronically elevated), IL-6, IL-8, CCL2/MCP-1, CCL5/RANTES, MMPs, and VEGF. Contributes to chronic inflammation, tissue remodeling, and fibrosis. Promotes "bystander effect".	[106, 112, 114]
Immune clearance	Efficiently cleared by immune cells like macrophages and NK cells.	Impaired clearance by dysfunctional NK cells and possibly macrophages. Leads to chronic accumulation of senescent cells.	[106, 112]
Cellular state & behavior	Stable cell cycle arrest, tissue remodeling, temporary presence, normal decidualization.	Irreversible growth arrest, resistance to apoptosis, sustained proliferation, impaired decidualization, fibrosis, chronic inflammation, pain.	[111, 114]
Impact on fertility	Essential for successful embryo implantation and pregnancy.	Contributes to recurrent implantation failure (RIF) and recurrent pregnancy loss (RPL), creates a hostile environment for embryo implantation. Associated with endometriosis-associated infertility.	[54, 106]

### Senescence and NK Cell Dysfunction

The critical balance between the generation and clearance of senescent cells appears disrupted in endometriosis. As mentioned, uterine NK (uNK) cells play a key role in clearing physiologically senescent decidual cells during the normal menstrual cycle, likely mediated by factors like IL-15 [82]. The established dysfunction of NK cells in endometriosis is characterized by reduced cytotoxicity [79]. Therefore, these cells may fail in their surveillance role not only against ectopic endometrial cells but also against senescent cells. This would lead to the accumulation of senescent cells, the persistence of their pro-inflammatory SASP, and the chronic inflammation and tissue disruption seen in the disease, further reinforcing the pathological cycle.

The dual nature of senescence in the endometrium is necessary transiently for implantation but detrimental when chronic, posing challenges for therapeutic interventions. Strategies like senolytics, which eliminate senescent cells, have shown promise in preclinical models for various age-related diseases and even for endometriosis [55, 113]. However, indiscriminate removal of all senescent cells in the endometrium could potentially disrupt physiological processes essential for fertility [16]. Therefore, therapeutic approaches may need to be more nuanced, perhaps targeting specific drivers of pathological senescence, modulating the harmful components of the SASP (senomorphics), or selectively targeting only the chronically persistent senescent cells.

## Fibrosis: The Scarring Component of Endometriosis

Fibrosis, characterized by the excessive deposition and accumulation of ECM components, primarily collagen, is a common endpoint of chronic inflammation and repetitive tissue injury across many organs [29]. In endometriosis, fibrosis manifests as scarring, dense adhesions between pelvic organs, and hardening of tissues containing ectopic implants. This contributes significantly to the disease’s morbidity, particularly pain and infertility [26].

### Mechanisms of Fibrogenesis in Endometriosis

The central cellular players in fibrosis are activated myofibroblasts, identifiable by their expression of α-SMA [85]. These cells are the primary source of the excessive ECM deposition in fibrotic lesions. Myofibroblasts in endometriosis can originate from several precursor cell types through transdifferentiation [86]. Resident stromal fibroblasts within the ectopic tissue or surrounding peritoneum can differentiate into myofibroblasts (FMT). Additionally, epithelial cells lining the endometriotic glands can undergo EMT, and endothelial cells of the lesion’s microvasculature can undergo EndMT. Both these processes yield cells with mesenchymal and fibrogenic characteristics [86]. EndMT appears particularly relevant in ovarian endometriomas, potentially linked to their angiogenic nature [112].

The activation and persistence of myofibroblasts are driven by a complex network of signaling molecules within the endometriotic microenvironment. The TGF-β signaling pathway is considered a master regulator of fibrogenesis in endometriosis, promoting myofibroblast differentiation and collagen synthesis [112]. Other growth factors like PDGF, often released from activated platelets found within lesions, also contribute [112]. Signaling pathways implicated in fibrosis in other contexts, such as the Wnt/β-catenin, Hedgehog, and Rho/ROCK pathways, may also be involved in endometriosis [112]. Chronic inflammation and repeated tissue injury from cyclical bleeding sustain myofibroblast activation and promote progressive ECM accumulation [22]. Immune cells like macrophages and potentially factors released from nerves within the lesions can also contribute pro-fibrotic signals [112].

### Consequences of Fibrosis in Endometriosis

The accumulation of dense, scar-like fibrotic tissue has significant clinical consequences:

- **Pain**: Fibrosis contributes directly to chronic pelvic pain, dysmenorrhea, and dyspareunia. The stiff, non-compliant fibrotic tissue can cause pain through mechanical distortion and reduced tissue elasticity. Furthermore, fibrotic lesions often exhibit increased innervation, and the fibrosis itself can entrap or irritate nerve fibers, leading to neuropathic pain components [112].
- **Infertility**: Extensive fibrosis forms adhesions that distort pelvic anatomy, encase ovaries, and block fallopian tubes, physically hindering oocyte release, pickup, transport, and fertilization [86]. Beyond mechanical obstruction, the altered tissue architecture and microenvironment within fibrotic lesions may also negatively impact ovarian function or endometrial receptivity.
- **Organ Dysfunction**: In cases of DIE, the invasive fibrotic lesions can infiltrate the walls of adjacent organs like the bowel, bladder, or ureters, leading to significant organ dysfunction, pain related to organ function (e.g., dyschezia, dysuria), and potentially requiring complex surgical interventions [112].

The recognition of fibrosis as a central component of endometriosis, potentially even warranting its redefinition as a “fibrotic condition” [26], marks a significant conceptual shift. This suggests that treatments targeting only hormones or inflammation may not resolve fibrosis-driven structural damage and symptoms. It underscores the need for anti-fibrotic therapies in endometriosis, potentially by targeting myofibroblast activation or ECM deposition, as seen in fibrotic diseases of the lung or liver [112]. The heterogeneity in myofibroblast origins (EMT/EndMT/FMT) and signaling pathways across different lesion types further suggests that understanding these specific mechanisms could pave the way for more targeted and effective anti-fibrotic interventions [85].

### The Triad Crosstalk: Iron Overload, Senescence, and Fibrosis in Endometriosis

The evidence presented thus far strongly suggests that iron overload, cellular senescence, and fibrosis are not independent processes occurring in endometriosis but are deeply intertwined. Together, they potentially form a self-perpetuating vicious cycle that drives disease progression and symptoms. Understanding the crosstalk between these three components is crucial for a comprehensive view of endometriosis pathogenesis.

### Iron Overload as a Driver of Senescence and Fibrosis

Iron overload appears capable of initiating and promoting both senescence and fibrosis. The generation of ROS via the Fenton reaction, catalyzed by excess labile iron, is a well-established mechanism for inducing cellular damage, including DNA damage [96]. Persistent DNA damage is a primary trigger for cellular senescence [112]. Studies in other systems have directly shown that iron accumulation can drive senescence and the expression of SASP factors [112]. This aligns with findings in endometriosis, where markers of oxidative stress and senescence are co-localized or associated [54]. Therefore, the iron-rich environment created by recurrent hemorrhage in endometriosis likely contributes directly to the induction of senescence in ectopic endometrial cells or surrounding peritoneal cells.

Similarly, iron overload is implicated in promoting fibrosis [99]. This may occur through several mechanisms. Iron-induced oxidative stress can directly damage tissues, triggering a chronic wound healing response that culminates in fibrosis [97]. Furthermore, iron overload has been shown to induce ferroptosis in specific endometrial stromal cell subpopulations, and this cell death process itself appears to promote a fibrotic response in ovarian endometriosis models [94]. Iron-laden macrophages, frequently observed within fibrotic endometriotic tissue, represent another critical link [22]. These macrophages, potentially skewed towards a pro-inflammatory M1 phenotype due to iron accumulation [93], are potent sources of both inflammatory cytokines and key pro-fibrotic mediators like TGF-β, thereby directly connecting iron processing by immune cells to the fibrotic process.

### Senescence as a Modulator of Iron Metabolism and Fibrosis

The hypothesized crosstalk is likely bidirectional; senescence may affect iron handling and promote fibrosis. Senescent cells often accumulate intracellular iron, largely in ferritin-bound lysosomes, yet maintain a labile iron pool that drives ROS production and SASP secretion [27]. This may establish a positive feedback loop as iron induces senescence, senescent cells accumulate more iron, and this reinforces both the senescent state and its harmful secretions. If this mechanism operates in endometriosis, senescent cells could act as persistent reservoirs of redox-active iron, exacerbating oxidative stress and inflammation even if the initial source of iron (e.g., acute bleeding) subsides.

Furthermore, the SASP secreted by senescent cells is inherently pro-fibrotic. Many SASP components, including pro-inflammatory cytokines like IL-1β, IL-6, and TNF-α, can stimulate myofibroblast activation and ECM production [55]. Additionally, SASP includes proteases like MMPs, which, while degrading some ECM components, also remodel the matrix in ways that can facilitate fibrosis progression and release matrix-bound growth factors (like TGF-β) [28]. Therefore, the accumulation of senescent cells in endometriosis directly contributes to the pro-fibrotic microenvironment.

### Fibrosis, Inflammation, and Iron Trapping

**Fig. 4:**
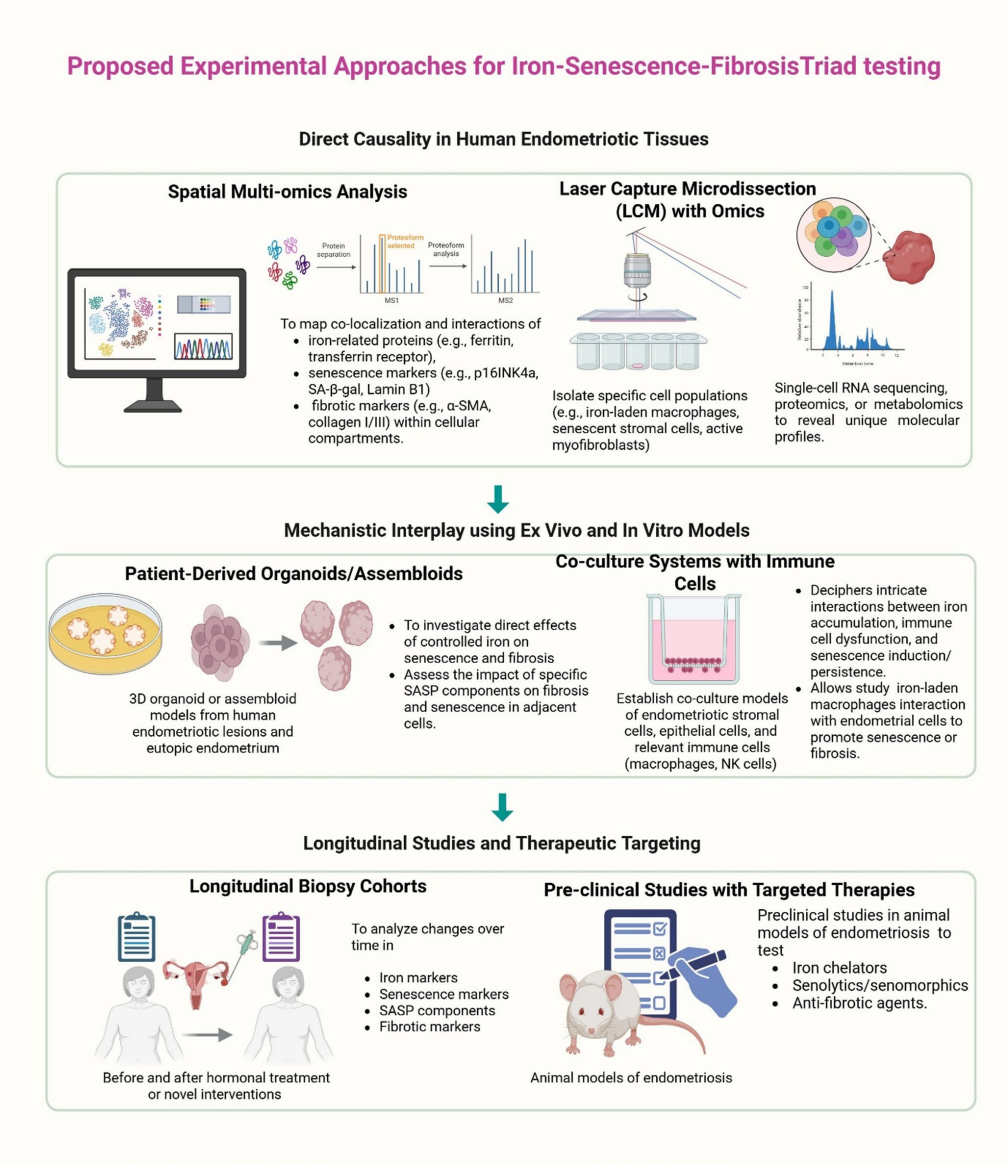
Proposed experimental approaches to test the “iron-senescence-fibrosis” triad in endometriosis. This diagram outlines a multi-faceted strategy to investigate the iron-senescence-fibrosis triad. Direct causality in human endometriotic tissues can be explored via spatial multi-omics analysis and laser capture microdissection (LCM) with Omics for detailed cellular profiling. For mechanistic interplay using ex vivo and in vitro models, patient-derived organoids/assembloids will probe iron’s effect on senescence and fibrosis, while co-culture systems with immune cells will decipher interactions between iron accumulation, immune dysfunction, and senescence. Lastly, longitudinal studies and therapeutic targeting involve longitudinal biopsy cohorts to track changes in patients over time and pre-clinical studies with targeted therapies in animal models to test interventions like iron chelators, senolytics, and anti-fibrotics

Based on literature-based evidence, it may be hypothesized that the dense fibrotic tissue associated with endometriosis may also contribute to the cycle. Extensive scarring and adhesion formation could potentially impede the normal clearance mechanisms within the peritoneal cavity, hindering the removal of inflammatory cells, cellular debris, and potentially trapping iron-rich fluid from micro-hemorrhages within. Key aspects of the hypothesized “iron-senescence-fibrosis” triad may be validated experimentally using the cutting-edge approaches highlighted in the Fig. 4.

### The Vicious Cycle

**Fig. 5:**
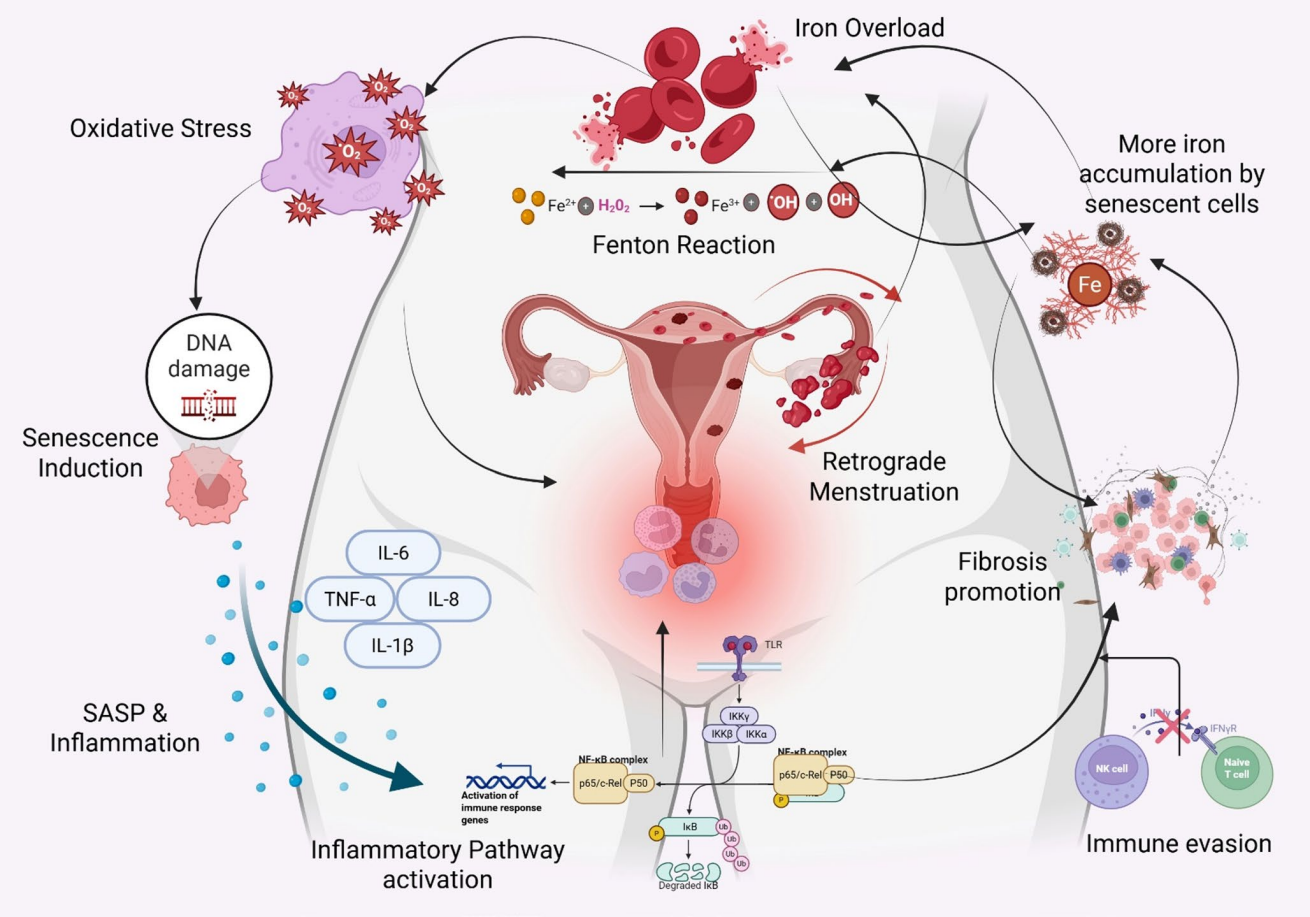
This illustration shows a self-perpetuating cycle underlying endometriosis. It begins with retrograde menstruation or lesion hemorrhage, leading to erythrocyte deposition and iron overload in the peritoneal cavity. Breakdown of red blood cells releases heme and iron, driving toxic labile iron accumulation, which catalyzes ROS generation via the Fenton reaction, causing oxidative stress. This oxidative damage induces cellular senescence, especially in ectopic endometrial and peritoneal cells. Senescent cells release a pro-inflammatory, pro-fibrotic SASP (e.g., IL-1β, IL-6, matrix metalloproteinases), which sustains chronic inflammation. Iron also activates inflammatory pathways like NF-κB. Meanwhile, immune dysfunction, notably impaired NK cell clearance, permits persistence of ectopic and senescent cells. These factors promote fibrosis, driven by fibroblast-to-myofibroblast conversion via TGF-β and excessive ECM deposition. Feedback loops worsen pathology with senescent cells accumulating more iron, fibrosis hampers clearance, and chronic inflammation perpetuates damage and micro-bleeding, reinforcing the cycle and disease progression

Synthesizing the roles of iron overload, cellular senescence, and fibrosis reveals a compelling model where these pathways intertwine to drive endometriosis pathogenesis. This integrated view, backed by published literature and emerging evidence, strongly suggests a self-perpetuating vicious cycle, where each component can initiate and amplify the others, ultimately fuelling disease progression. The proposed key steps in this pathogenic cycle are outlined in Fig. 5.

## Therapeutic Implications and Future Directions

The recognition of the interconnected roles of iron overload, cellular senescence, and fibrosis in endometriosis pathogenesis opens up new avenues for therapeutic intervention. Targeting the nodes and pathways within this triad holds promise for developing more effective strategies to manage the disease.

### Targeting Iron Overload

Since iron overload triggers oxidative stress, inflammation, senescence, and fibrosis, reducing pelvic iron accumulation presents a promising therapeutic strategy [22, 24]. Iron chelators, drugs that bind excess iron and facilitate its removal, have shown promise in preclinical models. Administration of deferoxamine (DFO) in mouse models of endometriosis effectively reduced iron load in lesions and peritoneal macrophages and significantly decreased the proliferative activity of the lesions [24]. Though clinical translation is needed, evidence suggests iron chelation may help prevent iron-induced damage and limit lesion growth, inflammation, and fibrosis. Further research should focus on optimizing delivery, assessing safety and efficacy, and exploring targets within iron transport and metabolism pathways.

### Targeting Cellular Senescence (Senotherapeutics)

The accumulation of senescent cells and their detrimental SASP provides another potential therapeutic target. Senotherapeutics encompass two main classes: senolytics, which selectively trigger apoptosis in senescent cells while sparing non-senescent tissue, and senomorphics, which suppress the SASP without necessarily killing the cells [55].

Preclinical studies have explored both approaches in contexts relevant to endometrial function or endometriosis. Senolytics like Dasatinib (D) and Quercetin(Q) have shown potential in slowing the aging of human endometrial stromal cells and mouse uterine tissue [15]. By inducing selective apoptosis only in senescent cells, senolytics can reduce the overall senescent cell burden and lessen the harmful effects of the SASP without disrupting the normal physiological processes that rely on transient senescence [112]. A small study on endometrial tissue from endometriosis patients showed that D, Q, and especially D + Q, reduced senescence and boosted decidualization markers, supporting further infertility research [112]. Navitoclax (ABT263) and the antibiotic Azithromycin (AZM), identified as having senolytic activity against fibroblasts, reduced the viability of senescent endometriotic stromal cells in vitro [55]. AZM, acting potentially as both a senolytic and senomorphic (suppressing IL-6 SASP factor), also showed beneficial effects in a mouse model of endometriosis [55]. Senomorphics like rapamycin and metformin have been shown to diminish the adverse effects of senescent endometrial stromal cells on decidualization and implantation models in vitro [17]. These agents modulate the SASP without killing the senescent cells. This approach aims to suppress the harmful pro-inflammatory and pro-fibrotic secretions of chronic senescent cells, thereby mitigating disease progression while leaving the cells themselves intact.

**Fig. 6:**
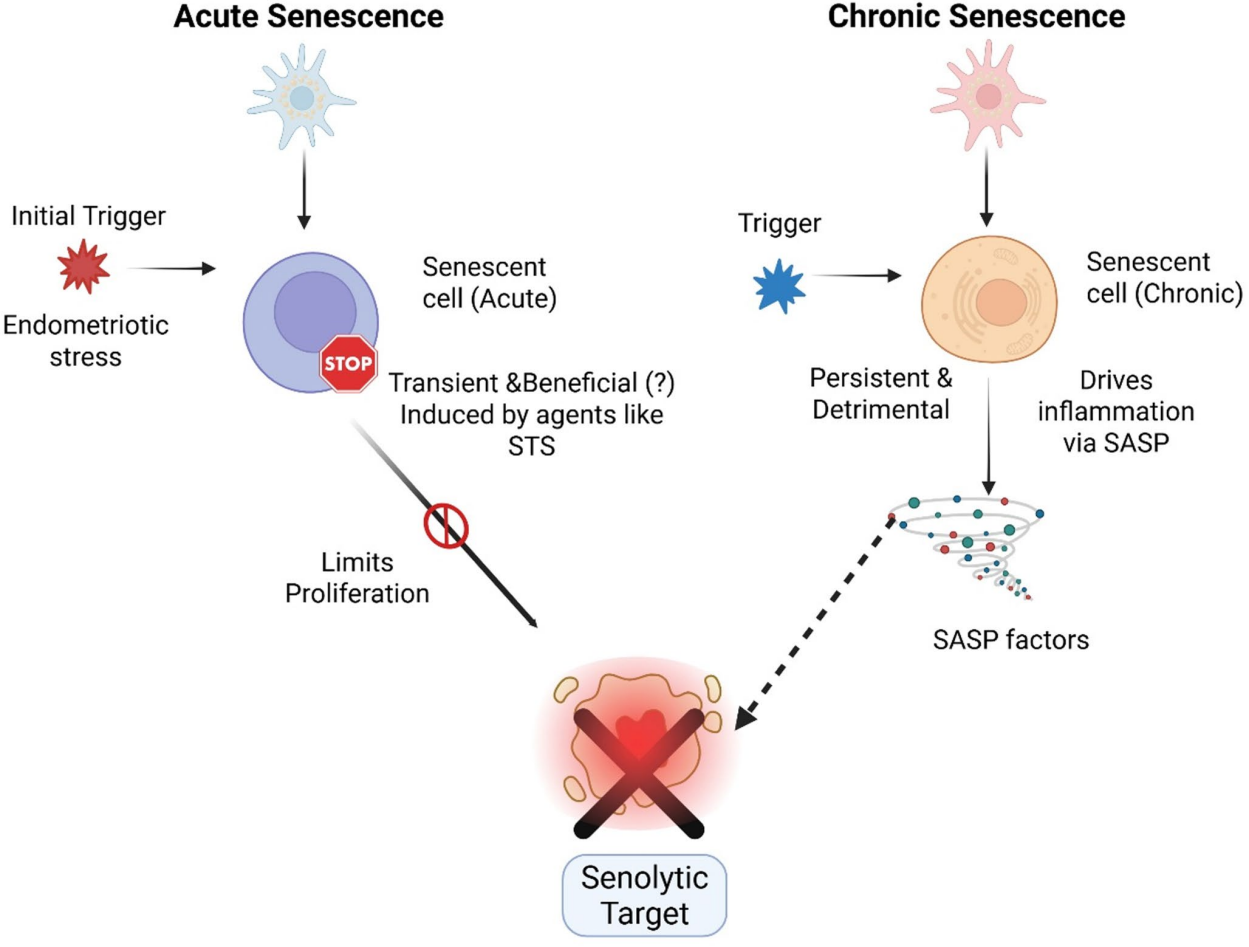
The dual role of cellular senescence in endometriosis pathogenesis. This figure differentiates between acute and chronic senescence in endometriosis. Acute senescence (left) is a transient, potentially beneficial response induced by stress or agents like STS, limiting proliferation and typically undergoing efficient immune clearance. Chronic senescence (right), driven by persistent triggers such as oxidative stress and iron overload, is detrimental due to the sustained release of pro-inflammatory and pro-fibrotic factors via the senescence-associated secretory phenotype (SASP). Unlike acute senescence, chronic senescent cells accumulate due to impaired immune clearance (e.g., by dysfunctional NK cells), actively driving disease pathology. Senolytic therapies aim to selectively target and eliminate these chronic, pathological senescent cells to reduce inflammation and fibrosis, while preserving beneficial acute senescence

However, caution is necessary when considering senotherapeutics for endometriosis, given the physiological role of transient senescence in endometrial receptivity and decidualization [16]. Indiscriminate elimination of all senescent cells could potentially impair fertility. Long-term usage of AZM is not practical as it may lead to antibiotic resistance problem and other associated toxicities. Moreover, many of the senolytic agents, like ABT263, are in the clinical trial stage. A study examining the effect of sodium tanshinone IIA sulfonate (STS) on deep endometriosis in mice showed that STS treatment reduces lesion weight, halts fibrogenesis, and improves hyperalgesia, seemingly by inducing cellular senescence [112]. This study highlights that senescence induction, rather than promoting proliferation, is associated with a reduction in lesion growth. This contradicts in removal of senescent cells could help in the management of endometriosis. Physiologically, transient and acute senescence is a beneficial process essential for tissue remodeling and repair. This is observed during normal embryonic development and wound healing, where senescent cells are generated and then quickly cleared by the immune system. In this context, STS-induced senescence may represent a form of acute stress response that triggers cell cycle arrest, thereby limiting the proliferation and growth of endometriotic lesions. Conversely, in pathological conditions like endometriosis, senescent cells evade immune clearance and accumulate chronically. This persistent presence leads to the continuous secretion of a pro-inflammatory and pro-fibrotic. Therefore, the goal of senolytic therapy is to eliminate these detrimental, chronically-persistent senescent cells rather than interfering with transient, beneficial senescence (Fig. 6). Senolytic therapy in endometriosis must selectively eliminate the detrimental, chronically persistent senescent cells that drive the pro-inflammatory and pro-fibrotic SASP, while preserving the transient, beneficial senescence essential for normal endometrial function. Future strategies must involve more selective senolytics, senomorphics to suppress harmful SASP factors, or targeting upstream triggers like iron overload that drive pathological senescence.

### Targeting Fibrosis

Directly targeting the fibrotic process offers another therapeutic avenue, particularly relevant for addressing pain and structural damage caused by adhesions and tissue scarring [87]. Given the shared mechanisms with other fibrotic diseases, there is potential for repurposing anti-fibrotic drugs approved or under investigation for conditions like idiopathic pulmonary fibrosis (IPF), liver cirrhosis, or systemic sclerosis [112]. Potential targets include the central TGF-β pathway, PDGF signaling, Rho/ROCK pathways involved in myofibroblast contraction, or focal adhesion kinase (FAK) signaling [86]. Preclinical studies in endometriosis models have explored targeting factors involved in fibrosis initiation, such as inhibiting platelet activation or mast cell activity, with some success [89]. However, translating these findings into effective clinical treatments for endometriosis requires further investigation, including understanding the dominant fibrotic pathways in different lesion types and stages.

#### Combined or Multi-Target Approaches

Due to the complex feedback within the iron-senescence-fibrosis triad, combination therapies targeting multiple pathways may be more effective than single-agent treatments. For instance, combining an iron chelator with a senomorphic agent or an anti-fibrotic drug could potentially disrupt the vicious cycle at multiple points, leading to a more comprehensive therapeutic effect. Designing and testing such combination strategies represents an important future direction. While promising, a multi-target strategy must also consider the potential risks associated with simultaneous targeting, particularly regarding its effects on normal tissue homeostasis.

### Patient Stratification for Personalized Therapy

**Fig. 7:**
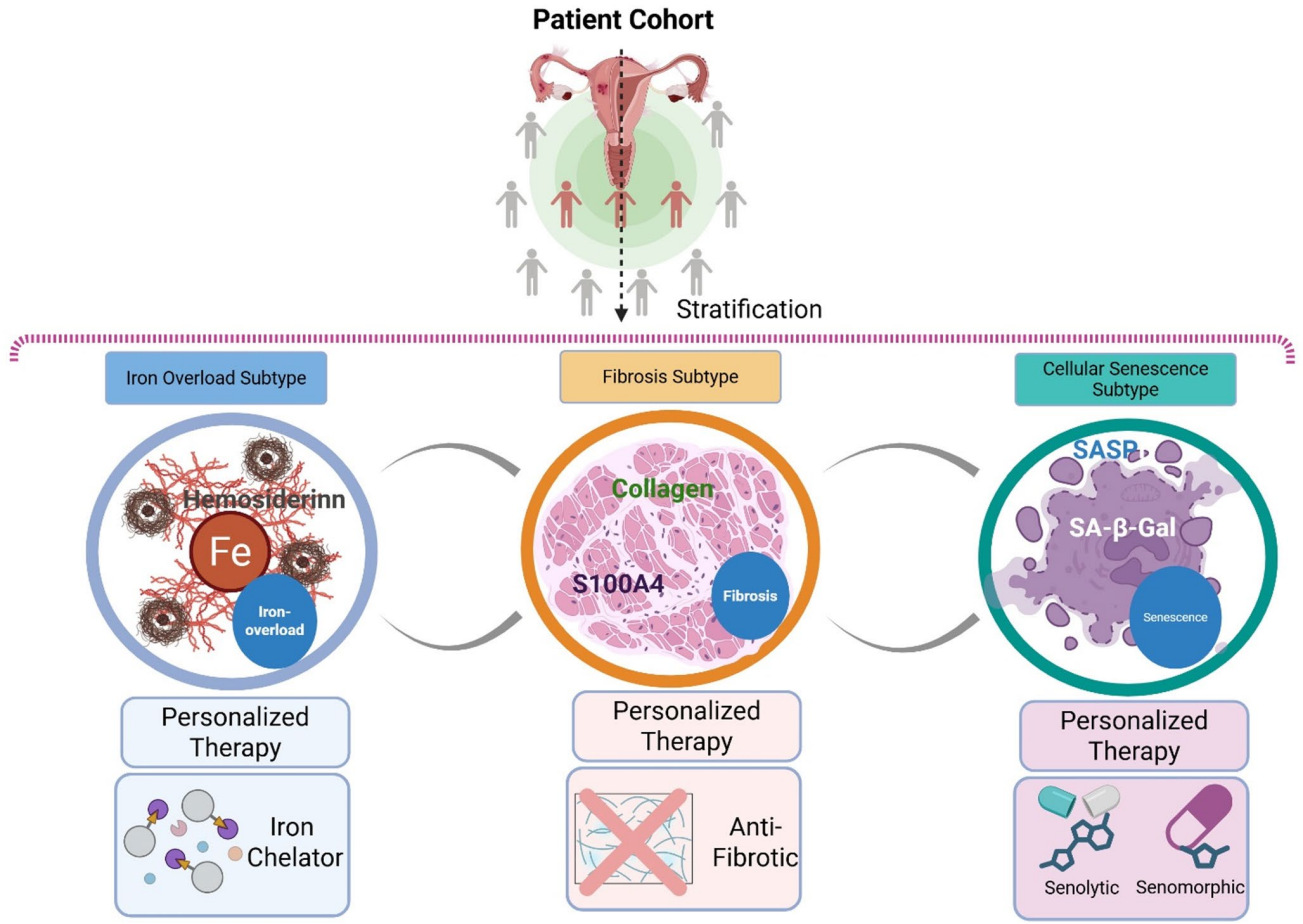
Patient stratification approach for personalized endometriosis therapy. This figure illustrates a patient stratification approach for personalized endometriosis therapy. Patients with this heterogeneous disease can be categorized into distinct subgroups based on specific biomarkers. The figure shows three key subtypes and their corresponding targeted therapies: Iron overload subtype: Characterized by markers like hemosiderin deposition, this group is an ideal candidate for iron chelator therapy. Fibrosis subtype: Identified by fibrotic markers such as collagen fibers, this group would benefit from anti-fibrotic drugs. Cellular senescence subtype: Defined by the presence of senescent cells and their harmful secretions (SASP), this group is a target for senolytics or senomorphics. This personalized approach aims to match the right treatment to the right patient, improving therapeutic effectiveness

Irrespective of the targeting approach, due to the heterogeneous nature of endometriosis, future therapeutic strategies should consider patient stratification based on potential biomarkers(Fig. 7). For instance, patients with high levels of peritoneal fluid ferritin or other markers of iron overload could be ideal candidates for iron chelation therapy. Patients with higher levels of fibrotic markers like S100A4 in blood or peritoneal fluid may be put on an anti-fibrotic regimen on priority. Similarly, individuals whose lesions show high expression of senescence markers like p16INK4a or SA-β-Gal activity might be prioritized for senolytic treatments, ensuring a more personalized and effective approach.

## Conclusion

**Fig. 8:**
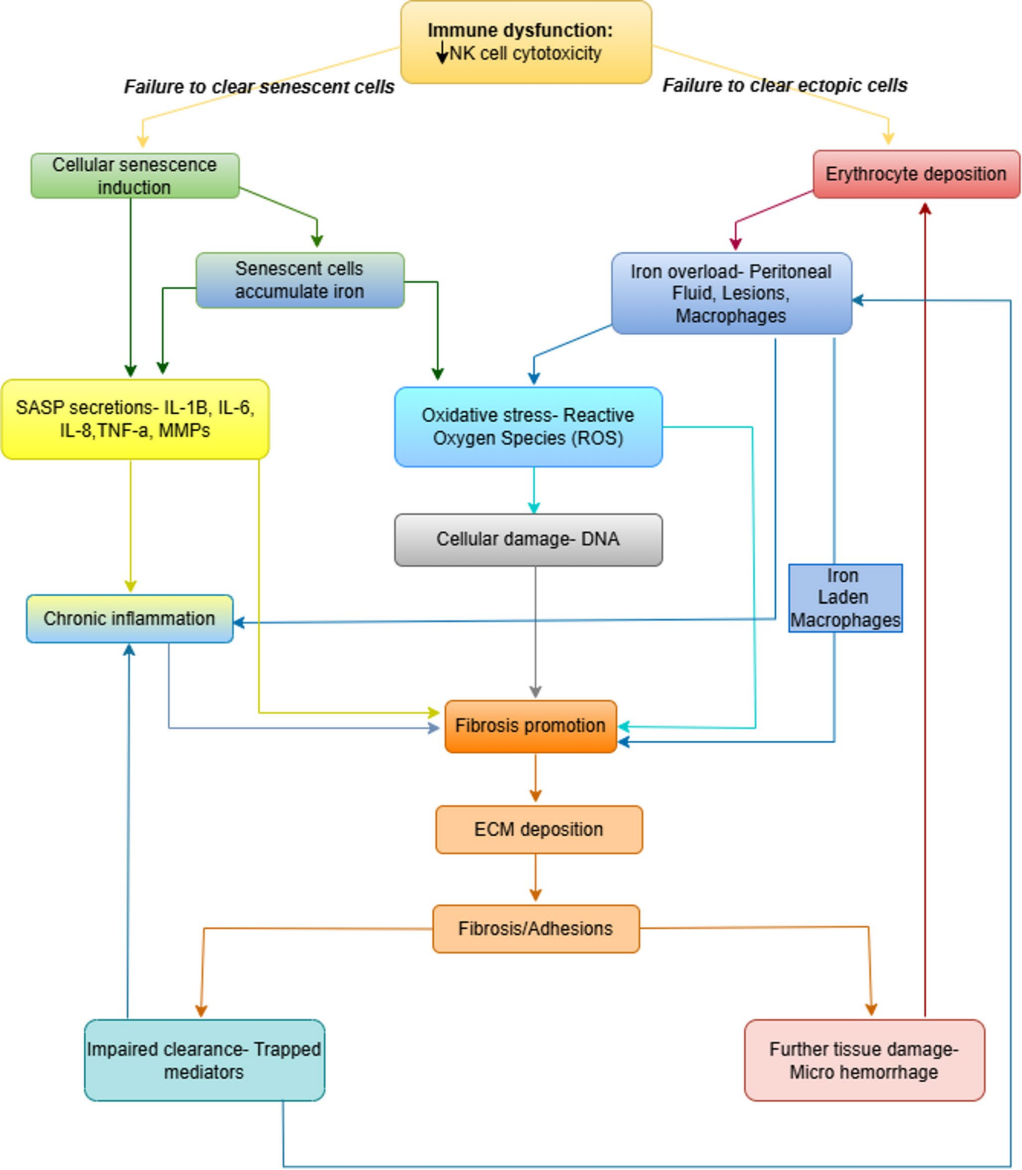
This schematic illustrates the hypothesized pathological axis linking cellular senescence, iron overload, and fibrosis in endometriosis. In non-endometriosis conditions, senescent cells exhibit a transient Senescence-Associated Secretory Phenotype (SASP), releasing pro-regenerative cytokines (e.g., IL-1, IL-2, IL-1β), which support tissue repair and regeneration. Iron (Fe) levels remain balanced, minimizing oxidative stress. In contrast, endometriosis is marked by persistent SASP, characterized by the chronic secretion of pro-inflammatory cytokines (e.g., IFNγ, TNFα) and iron-induced oxidative stress, leading to impaired immune clearance of senescent cells, fibrosis, chronic inflammation, and pain. The imbalance between transient and persistent SASP underscores the pathological transition from normal tissue repair to fibrosis and disease persistence

The pathogenesis of endometriosis is increasingly understood to extend beyond the classical pillars of ectopic endometrial tissue, hormonal dependence, and inflammation. This review synthesizes compelling evidence implicating a detrimental triad of interconnected biological processes, iron overload, cellular senescence, and fibrosis, as key contributors to the development, maintenance, and clinical manifestations of the disease (Fig. 8). However, the direct, mechanistic interplay between all three components is often inferred from studies in other tissue systems rather than being directly demonstrated in endometriosis. Therefore, a major limitation is the need for more functional studies that specifically test these relationships within human endometriotic tissue. Future research should leverage advanced techniques like spatial multi-omics analysis and patient-derived organoids/assembloids to directly validate how iron accumulation drives senescence and fibrosis in this disease, which is crucial for translating this hypothesis into clinical practice.

The review derives robust evidence from human tissues, in vitro studies, and animal models that support the iron-senescence-fibrosis triad in endometriosis. However, human studies often show associations between senescence and endometriosis, rather than proving causality, and small, heterogeneous cohorts limit the applicability of the findings. In vitro systems, while insightful, may not fully recapitulate the in vivo microenvironment. The interplay between iron, senescence, and fibrosis in endometriosis, though biologically plausible, is complex and sometimes inferred from other systems. Therefore, functional studies targeting pathological pathways will be necessary for further validation. Interventions aimed at mitigating iron toxicity (e.g., chelation), selectively clearing pathological senescent cells or modulating their SASP (senotherapeutics), or directly inhibiting fibrotic pathways hold potential for developing more effective, non-hormonal treatments. However, more research is needed to fully understand how this process works in human endometriosis, considering variations between patients and lesion types. The goal is to develop safe and effective treatments to ease the burden of this condition.

## Data Availability

Data sharing is not applicable to this article, as no datasets were generated or analyzed during the current study.
